# Quantitative Susceptibility Mapping Values Quantification in Deep Gray Matter Structures for Relapsing‐Remitting Multiple Sclerosis: A Systematic Review and Meta‐Analysis

**DOI:** 10.1002/brb3.70093

**Published:** 2024-10-17

**Authors:** Sana Mohammadi, Sadegh Ghaderi, Farzad Fatehi

**Affiliations:** ^1^ Neuromuscular Research Center, Department of Neurology, Shariati Hospital Tehran University of Medical Sciences Tehran Iran; ^2^ Department of Neuroscience and Addiction Studies, School of Advanced Technologies in Medicine Tehran University of Medical Sciences Tehran Iran; ^3^ Neurology Department University Hospitals of Leicester NHS Trust Leicester UK

**Keywords:** deep gray matter, multiple sclerosis, quantitative susceptibility mapping, relapsing‐remitting multiple sclerosis

## Abstract

**Background/Objectives:**

This systematic review and meta‐analysis aimed to investigate the role of magnetic susceptibility (χ) in deep gray matter (DGM) structures, including the putamen (PUT), globus pallidus (GP), caudate nucleus (CN), and thalamus, in the most common types of multiple sclerosis (MS) and relapsing‐remitting MS (RRMS), using quantitative susceptibility mapping (QSM).

**Methods:**

The literature was systematically reviewed up to November 2023, adhering to PRISMA guidelines. This study was conducted using a random‐effects model to calculate the standardized mean difference (SMD) in QSM values between patients with RRMS and healthy controls (HCs). Publication bias and risk of bias were also assessed.

**Results:**

Nine studies involving 1074 RRMS patients with RRMS and 640 HCs were included in the meta‐analysis. The results showed significantly higher QSM (χ) values in the PUT (SMD = 0.40, 95% confidence interval [CI] = 0.22–0.59, *p* = .000), GP (SMD = 0.60, 95% CI = 0.50–0.70, *p* = .00), and CN (SMD = 0.40, 95% CI = 0.15–0.66, *p* = .005) of RRMS patients compared to HCs. However, there were no significant differences in the QSM values in the thalamus between patients with RRMS and HCs (SMD = −0.33, 95% CI −0.67–0.01, *p* = .026). Age‐ and sex‐based subgroup analysis demonstrated that younger patients (< 40 years) in the PUT, GP, and CN groups and larger male populations (> 25%) in the PUT and GP groups had more significant χ. Interestingly, thalamic QSM values were found to decrease in RRMS patients over 40 years of age and in higher male populations. Sex‐based subgroup analysis indicated higher iron levels in the PUT and GP of RRMS patients regardless of sex. QSM values were higher in certain brain regions (PUT, GP, and CN) during the early stages (disease duration < 9.6 years) of RRMS, but lower in the thalamus during the later stages (disease duration > 9.6 years) than HCs.

**Discussion/Conclusion:**

QSM may serve as a biomarker for understanding χ value alterations such as iron dysregulation and its contribution to neurodegeneration in RRMS, especially in the basal ganglia nuclei including PUT, GP, and CN.

## Graphical abstract

The study revealed that iron accumulation was significantly increased in the putamen, globus pallidus, and caudate nucleus of patients with relapsing‐remitting multiple sclerosis (RRMS). However, quantitative susceptibility mapping (QSM) values did not show significant differences in the thalamus. Interestingly, younger RRMS patients (< 40 years) exhibited greater iron deposition in the putamen, globus pallidus, and caudate nucleus, while male populations (> 25%) showed higher iron deposition in the putamen and globus pallidus. Furthermore, QSM values were elevated in certain brain regions (putamen, globus pallidus, and caudate nucleus) during the early stages of RRMS but decreased in the thalamus during the later stages compared to healthy controls. These findings suggest that QSM could be a valuable biomarker for detecting neurodegeneration in RRMS.

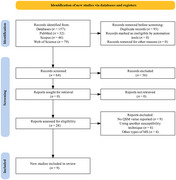

## 1 Introduction

Multiple sclerosis (MS) is a chronic inflammatory disease with neurodegeneration that affects the central nervous system (CNS) owing to immune‐mediated damage (Lury et al. 2023; Mey, Mahajan, and DeSilva 2023). It presents unique complexities in quantification and diagnosis (Lury et al. 2023). Between 2013 and 2020, the global number of patients diagnosed with MS increased from 2.3 million to 2.8 million (Walton et al. 2020).

The four acknowledged MS phenotypes include clinically isolated syndrome (CIS), relapsing‐remitting MS (RRMS), and primary progressive MS (PPMS), which involve a gradual neurological decline from the beginning, and secondary progressive MS (SPMS), which is a progressive disease that occurs after an initial course of RRMS (Lublin et al. 2014). Typically, RRMS starts in 85% of cases (Klineova and Lublin 2018), where inflammation in the CNS caused by the adaptive and innate components of the immune system leads to myelin destruction and quick neurological decline (Abdelhak et al. 2018; Pogoda‐Wesołowska et al. 2023). This results in a distinct episode of acute neurological dysfunction, followed by complete or partial relief and eventual disappearance of symptoms. Nearly 80% of patients with RRMS develop SPMS within 20 years of onset. In 50% of the patients, SPMS occurs within 10 years of the first episode (Statsenko et al. 2023; Ziemssen et al. 2022). Additionally, the accumulation of iron in the basal ganglia, predominantly in the putamen (PUT) and white matter (WM) lesions of patients with RRMS, has been linked to disease progression and neurological disability (Bergsland et al. 2017; Haider et al. 2014; Stüber, Pitt, and Wang 2016).

Iron plays a crucial role in brain function, and maintaining proper iron homeostasis is essential for cognitive processes (Singh et al. 2014). However, excess iron can lead to oxidative stress, inflammation, and neurotoxicity, resulting in neuronal damage and dysfunction (Stankiewicz, Neema, and Ceccarelli 2014). Therefore, it is vital to maintain a balanced iron level in the brain to ensure optimal health and performance (Lury et al. 2023). Accurately measuring the amount of iron in the brain is a significant advancement in the understanding and treatment of neurodegenerative diseases (Ghaderi et al. 2023; Li et al. 2023). Iron deposits containing Fe^3+^ are paramagnetic and have a powerful dipole that distorts the magnetic field and reduces T2^*^ relaxation time constants (Lury et al. 2023). Consequently, iron deposits can be observed and quantified using iron‐sensitive magnetic resonance imaging (MRI) techniques such as quantitative T2* imaging, susceptibility‐weighted imaging (SWI), and quantitative susceptibility mapping (QSM) (Ghaderi et al. 2024; Harada et al. 2022; Lury et al. 2023; Mohammadi, Ghaderi, and Fatehi 2024). QSM is a post‐processing solution owing to its noninvasive nature and quantitative capabilities (Ghaderi et al. 2023; Ravanfar et al. 2021). The production of a spatial map (spatial distribution of magnetic susceptibility [χ]) of magnetic susceptibilities is critical for determining iron concentration or demyelination within deep gray matter (DGM) structures (Fiscone et al. 2023). QSM provides an improved signal‐to‐noise and contrast‐to‐noise ratio for structures compared to their magnitude counterparts(Fujiwara et al. 2017; Lury et al. 2023; Madden and Merenstein 2023; Mohammadi, Ghaderi, and Fatehi 2024). Most significantly, χ directly reflects the molecular composition and cellular architecture of a tissue (Liu et al. 2015).

The DGM comprises subcortical components of the brain, including the thalamus and basal ganglia, PUT, globus pallidus (GP), and caudate nucleus (CN) (Fujiwara et al. 2017). It is transported to the brain through both transferrin‐dependent and transferrin‐independent pathways and stored as ferritin (Mills et al. 2010). Iron rims around chronic active lesions are frequently observed in the brains of patients with lesions (Weber et al. 2022). The presence of both DGM iron and iron rims indicates persistent inflammation caused by activated microglia, resulting in inability to remyelinate and tissue damage (AlTokhis et al. 2020; Lury et al. 2023). These markers were observed early in the course of RRMS (Lury et al. 2023).

The presence of χ alterations in certain DGM structures has been suggested to have a role in the development of MS (Haacke et al. 2009; Haider et al. 2014; Pontillo et al. 2021, 2023). Higher levels of iron in the DGM can be observed even in the early stages of RRMS than in HCs, particularly in the PUT and GP (Lury et al. 2023). The findings regarding iron levels in the thalamus and pulvinar nucleus are variable (Lury et al. 2023). However, the exact role of χ alterations such as asiron deposition in MS remains unclear. Therefore, χ alterations in DGM structures are increasingly recognized as an important aspect of the multidimensional pathology of MS, particularly in cases of the RRMS variant, as iron concentration or demyelination in these areas is a characteristic of the disease (Elkady et al. 2017; Lury et al. 2023; Ropele et al. 2014).

Iron deposition in the DGM may occur independently but simultaneously with demyelination, and these deposits contribute to cognitive impairment and disability in MS (Lury et al. 2023). A recent systematic review by De Lury et al. (2023) suggested that DGM iron could be a promising biomarker for understanding the pathophysiology of MS. Therefore, it is crucial to conduct a systematic review and meta‐analysis to synthesize the current literature on QSM studies on the DGM structures of RRMS. This knowledge would help to determine the usefulness of QSM as a biomarker in RRMS and provide insights into the extent and distribution of iron deposition, further enhancing our understanding of the role of iron in neurodegeneration.

## 2 Methods

### 2.1 Search Strategy

The study followed the guidelines of the Preferred Reporting Items for Systematic Reviews and Meta‐Analyses (PRISMA) (Page et al. 2021), and it examined PubMed, Scopus, and Web of Sciences databases in a systematic manner to find relevant studies published until November 2023. The search terms used were “quantitative susceptibility Mapping” OR “QSM” AND “relapsing‐remitting multiple sclerosis” OR “multiple sclerosis” AND “basal ganglia” OR “striatum” OR “caudate nucleus” OR “putamen” OR “globus pallidus” OR “substantia nigra pars reticulata” OR “subthalamic nucleus” OR “thalamus” OR “red nucleus” OR “substantia nigra pars compacta” OR “substantia nigra.” The search strategy was customized for each database (Table S1).

### 2.2 Study Selection and Screening

The authors included only studies published in peer‐reviewed English journals and reported QSM values/χ values in the DGM (PUT, GP, CN, and thalamus) of RRMS patients. In addition, relevant publications were manually searched from the reference lists of eligible studies. The authors excluded case reports, conference abstracts, review articles, animal studies, and nonoriginal studies. Furthermore, studies that used other quantitative MRI methods, such as R2^*^, and did not specifically mention QSM values in the DGM were also excluded. All studies retrieved from the databases were imported into the Endnote software (www.endnote.com), and duplicates were removed to prepare a list of selected studies. Two reviewers independently reviewed the titles, abstracts, and full texts to identify studies that used QSM to measure iron levels in the DGM of RRMS patients. Based on the eligibility criteria of our review, full‐text articles were screened using the articles selected from the primary screening. The reviewers reviewed the articles and excluded those that did not meet the eligibility criteria. Reasons for exclusion were noted. Conflicts between the two investigators in selecting the studies were resolved during the screening process, and the final studies were selected through consensus.

### 2.3 Data Extraction and Risk of Bias Assessment

The data from each study included in the research were collected by the authors. The main data extraction process was divided into several sections that met the eligibility criteria. The focus was on the study's characteristics, such as the first author's name and publication year, country of the first author's affiliation, field strengths, coil channels, subjects (patients and HCs), and DGM iron QSM values. To evaluate the quality and potential biases regarding selection, comparability, and outcome, both authors used the modified Newcastle–Ottawa scale (NOS) based on the Ottawa checklist for cross‐sectional studies (Ottawa Hospital Research Institute n.d.; Wells et al. 2000). The quality of studies can be determined by the final score on the NOS checklist, which ranges from 0 to 10. Scores of 9–10 indicate very good quality, scores 7–8 indicate good quality, scores 5–6 indicate satisfactory quality, and scores 0–4 indicate unsatisfactory quality. For this review, we modified the NOS, as in two previous meta‐analyses (Herzog et al. 2013; Parasuaraman et al. 2023).

### 2.4 Statistical Analysis

Meta‐analysis was conducted using Stata version 17 (StataCorp, College Station, TX, USA). Data extraction is performed to gather sufficient data for a specific region. The standardized mean difference (SMD) was used to analyze iron levels between the patient and control groups. Cohen's *d* cut‐off values (0.2, 0.5, and 0.8) were applied (Cohen 1988). A random effects model was used for the analysis. Heterogeneity was evaluated using *I* ^2^ statistics (Higgins and Thompson 2002), with values greater than 50% indicating moderate‐to‐high heterogeneity. Subgroup analyses were also conducted based on age (< 40 years, > 40 years), sex (male percentage of studies < 25%, > 25%), and disease duration (< 9.6 years, > 9.6 years) for the four selected DGM (Table 2 and Figures S1–S12). Owing to the limited number of studies, we did not perform a subgroup analysis for any other features. Additionally, sensitivity analyses were conducted to assess the influence of a study with exceptionally high or low results (Figure S13).

### 2.5 Publication Bias

To examine publication bias, both visual and quantitative analyses were performed. Funnel plots were used for visual analysis, and Egger's regression test was used for quantitative analysis (Egger et al. 1997). A significant publication bias was considered if the *p* value was less than .05. Further analysis was conducted using linear regression analysis, which considered both the intercept and slope parameters. The calculation was performed using Equation (1), where *i* represents the number of studies, which is equal to *r*. The standardized estimate is represented by *yi*, the precision of studies is represented by *xi*, and the error terms are represented by *ϵi*.

(1) yi=α+βxi+εi.

## 3 Results

### 3.1 Summary of Results

**FIGURE 1:**
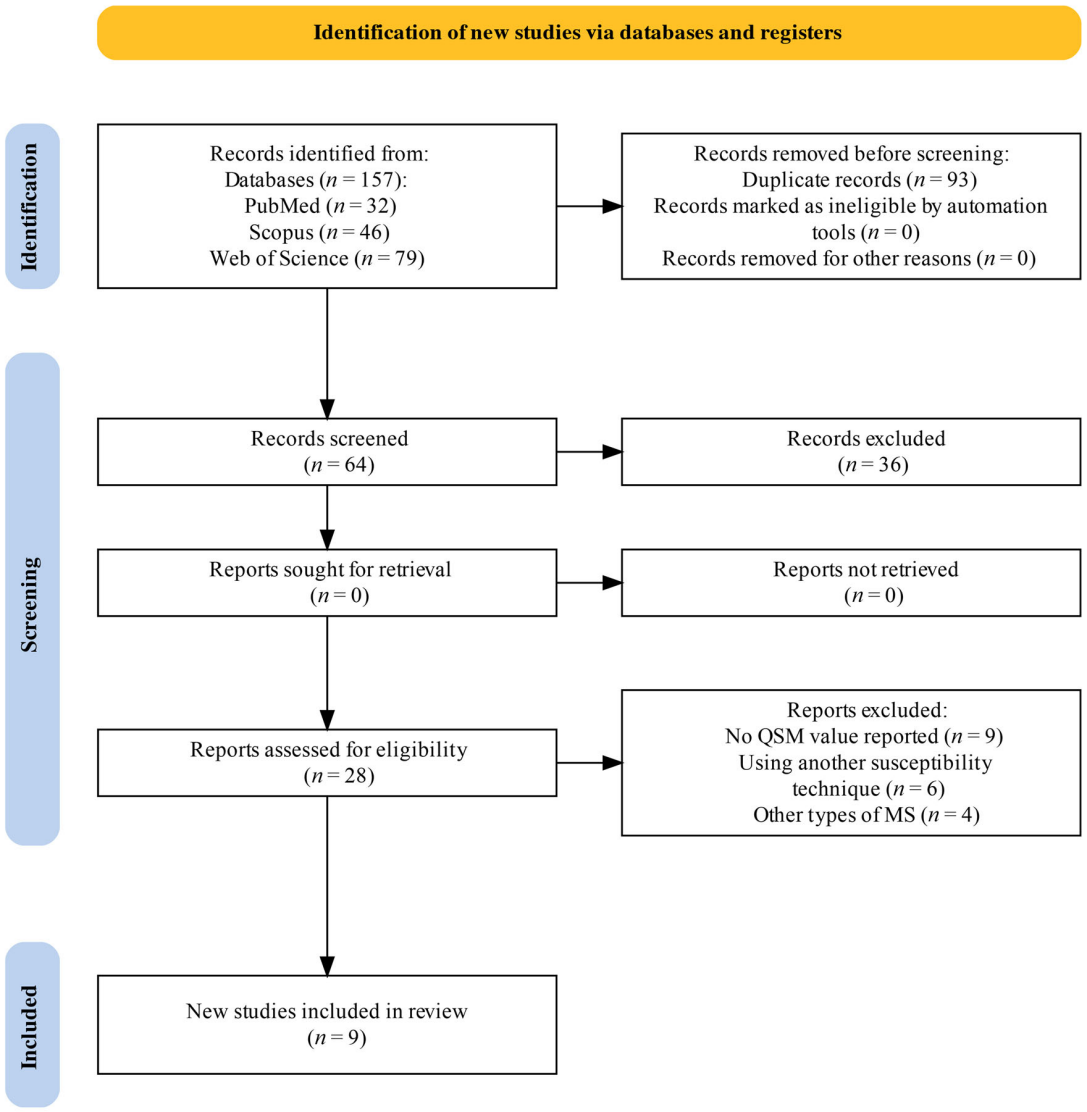
PRISMA flow diagram for systematic review.

A meta‐analysis was conducted, including nine studies (Figure 1). These studies involved 1074 patients with RRMS and 640 healthy controls (HCs). The magnetic field strengths used in the studies were 3T (four studies), 4.7T (three studies), and 1.5T (two studies). The reported QSM values and key characteristics of the included studies are listed in Table 1.

**TABLE 1:** Summary of included studies on deep gray matter structures in relapsing‐remitting multiple sclerosis.

Main characteristics				Subjects					QSM value (ppb) ± SD							
Study	Country	FS	Coil	Patients	Disease duration (years)	Mean age (patients)	Gender (M)	Controls	PUT (P)	PUT (C)	GP (P)	GP (C)	CN (P)	CN (C)	Th (P)	Th (C)
Cobzas et al. (2015)	Canada	4.7	NR	37	5.28	35.64	6	37	108 ± 18	93 ± 20	172 ± 20	150 ± 32	106 ± 14	93 ± 20	73 ± 13	63 ± 13
Elkady et al. (2017)	Canada	4.7	4	41	6.2	39.00	7	75	110 ± 16	102 ± 13	207 ± 23	192 ± 22	107 ± 14	101 ± 12	62 ± 12	62 ± 9
Elkady et al. (2019)	Canada	4.7	NR	25	10.7	37.30	3	25	105 ± 17.5	97.5 ± 18.7	207.5 ± 19.3	195.3 ± 27	109.2 ± 16.3	104.8 ± 17	63.6 ± 13.8	62.5 ± 10.1
Langkammer et al. (2013)	USA	3	12	42	7.3	34.60	14	23	73 ± 28	53 ± 17	179 ± 19	163 ± 31	64 ± 15	49 ± 10	4 ± 9	5 ± 6
Hagemeier, Zivadinov, et al. (2018)	USA	3	8	98	NR	NR	NR	40	50.5 ± 15.6	48.7 ± 11.8	121.7 ± 24.4	103.3 ± 15.7	43.3 ± 13.5	36.4 ± 8.8	−5.5 ± 7.9	−0.4 ± 6.7
Hagemeier, Ramanathan, et al. (2018)	USA	3	8	261	NR	NR	NR	150	50.6 ± 14.7	48.3 ± 15.55	120.9 ± 23.75	108.1 ± 21.2	43.7 ± 11.6	37.2 ± 10.4	−6.15 ± 8.65	−0.7 ± 7.05
Burgetova et al. (2017)	Czech Republic	1.5	NR	80	12.4	46.90	48	20	24.3 ± 10.7	20.3 ± 5.4	71.9 ± 14	66.7 ± 8.5	35 ± 9.6	32.6 ± 9.5	2.6 ± 4.9	5.9 ± 3.3
Pudlac et al. (2020)	Czech Republic	1.5	NR	40	14.1	47.90	8	20	30.3 ± 11.7	30.7 ± 7.4	83.6 ± 16.1	83.7 ± 11.1	39.3 ± 10.8	41.1 ± 10.1	10.8 ± 5.6	16.1 ± 8.2
Zivadinov et al. (2018)	USA	3	8	452	11.3	43.10	132	250	55.8 ± 14.1	47.9 ± 15.2	121.8 ± 24.9	107.3 ± 20.1	43.6 ± 11.9	44.5 ± 12.1	−6.4 ± 8.1	−1.1 ± 6.3

Abbreviations: C, control; CN, caudate nucleus; FS, field strength; GP, globus pallidus; HC, healthy control; NR, not reported; P, patient; PUT, putamen; QSM, quantitative susceptibility mapping; QSM value → parts per billion (ppb); Th, thalamus.

### 3.2 Geographic Distribution

Analysis of the geographical distribution revealed a higher number of studies from American countries, particularly North America, with four studies from the United States and three from Canada. Additionally, two studies were from the Czech Republic in Europe. However, no such studies have been conducted in Asia, Africa, or Oceania.

### 3.3 Meta‐Analysis

**FIGURE 2:**
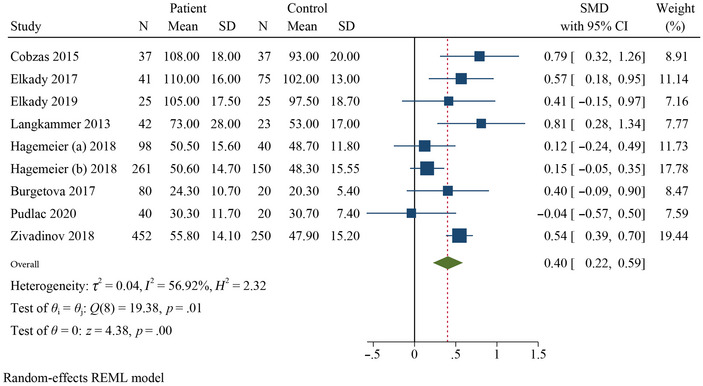
Forest plot comparing the mean susceptibility of QSM values for putamen in RRMS patients compared to HCs.

**FIGURE 3:**
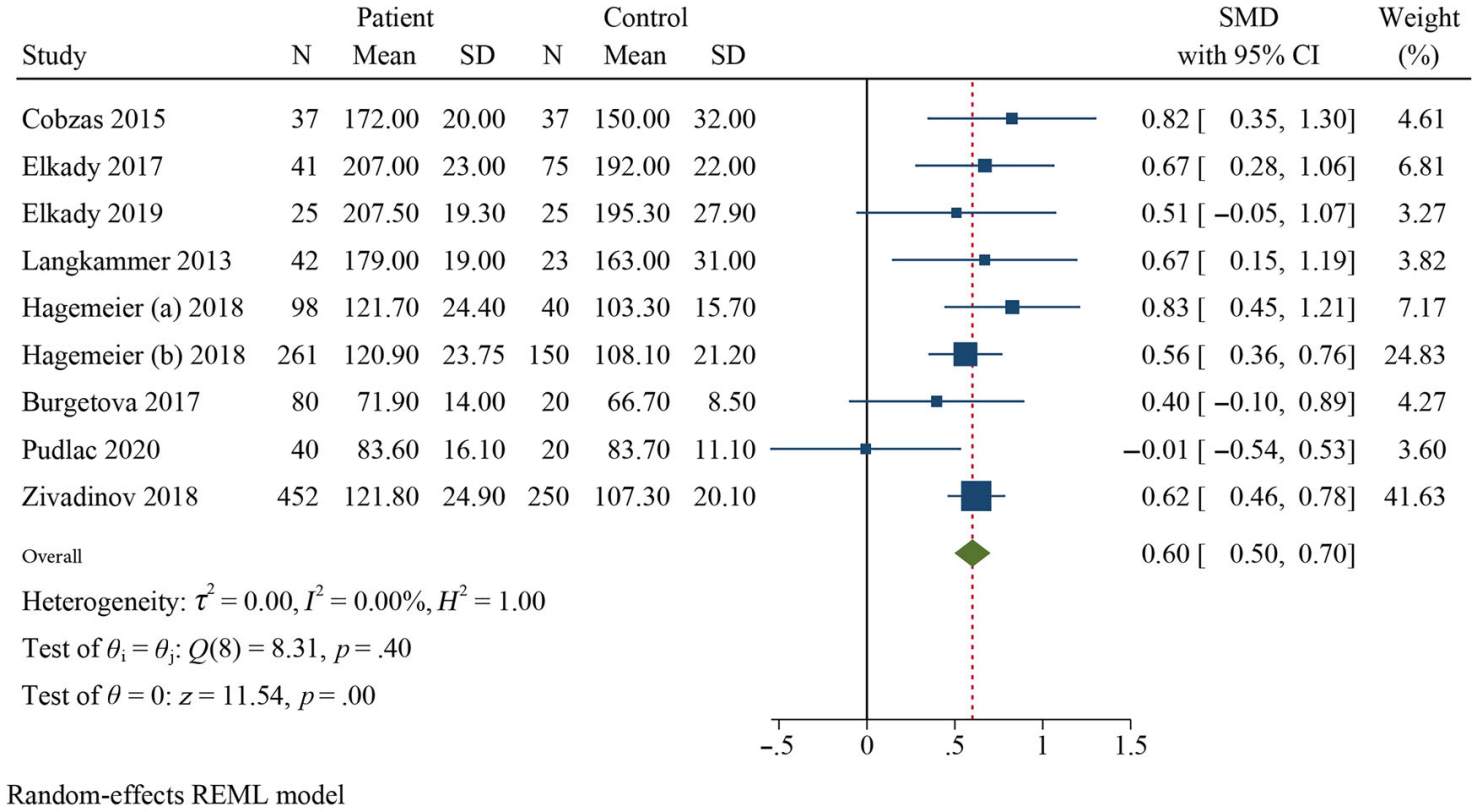
Forest plot comparing the mean susceptibility of QSM values for globus pallidus in RRMS patients compared to HCs.

**FIGURE 4:**
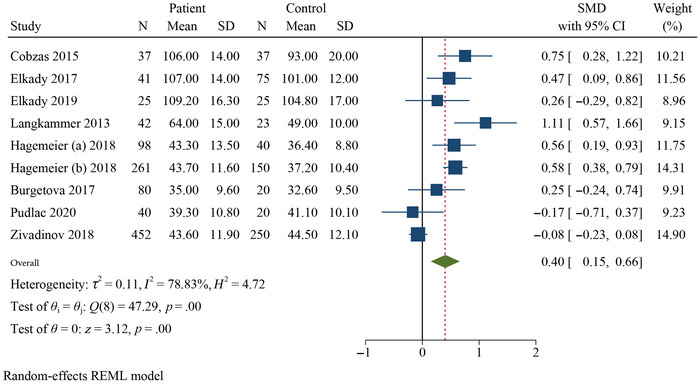
Forest plot comparing the mean susceptibility of QSM values for caudate nucleus in RRMS patients compared to HCs.

**FIGURE 5:**
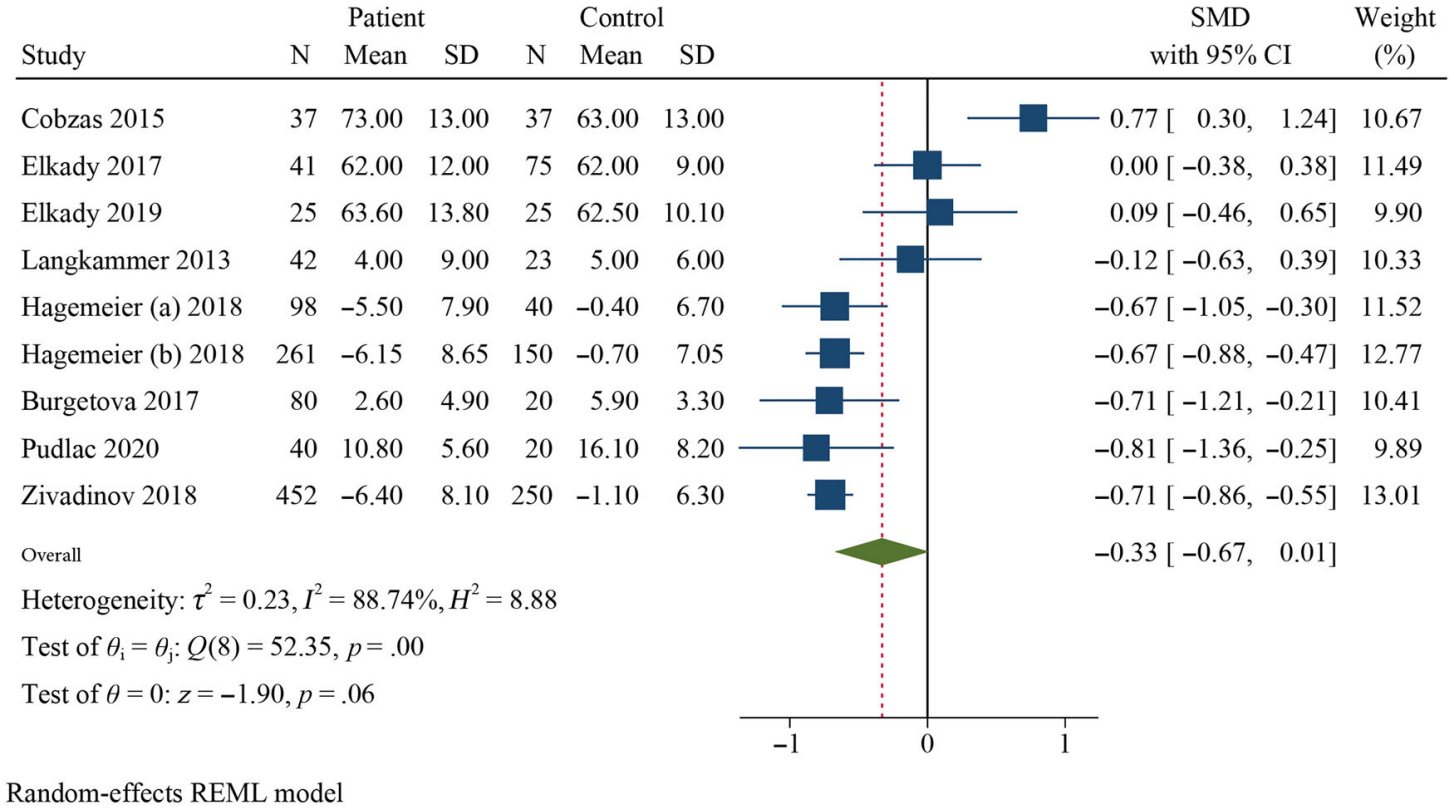
Forest plot comparing the mean susceptibility of QSM values for thalamus in RRMS patients compared to HCs.

Table 2 presents a summary of the results of the meta‐analysis and subgroup analysis. The meta‐analysis revealed that the QSM values of the pooled SMDs for the PUT (SMD = 0.40, 95% confidence interval [CI] = 0.22–0.59, *p* = .000, *I* ^2^ = 56.92%, *k* = 9, *n* = 1074), GP (SMD = 0.60, 95% CI = 0.50–0.70, *p* = .000, *I* ^2^ = 0.00%, *k* = 9), and CN (SMD = 0.40, 95% CI = 0.15–0.66, *p* = .005, *I* ^2^ = 78.33%, *k* = 9) were significantly higher in RRMS patients than in HCs (Figures 2, 3, 4). Additionally, the meta‐analysis showed that the QSM values of pooled SMD in the thalamus (SMD = −0.33, 95% CI: −0.67, 0.01, *p* = .026, *I* ^2^ = 88.74%, *k* = 9) did not show significant differences (negative effect) between RRMS patients and HCs, indicating a decreased χ in this DGM structure (Figure 5). Subgroup analyses were conducted to investigate the sources of heterogeneity and to assess the impact of different factors on the QSM values of the DGM (Table 2 and Figures S1–S12).

**TABLE 2:** Summary of meta‐analysis and age and sex subgroup analysis results for DGM of RRMS.

				Heterogeneity	
Overall	*k*	SMD (with 95% CI)	*p* value	*P*_heterogeneity_	*I* ^2^ %
PUT	9	0.40 (0.22, 0.59)	.000	0.01	56.92
GP	9	0.60 (0.50, 0.70)	.000	0.00	0.00
CN	9	0.40 (0.15, 0.66)	.005	0.00	78.33
Th	9	−0.33 (−0.67, 0.01)	.026	0.06	88.74
Age subgroup					
PUT: < 40	4	0.64 (0.41, 0.88)	.000	0.67	0.00
PUT: > 40	3	0.38 (0.06, 0.70)	.021	0.12	53.51
GP: < 40	4	0.68 (0.44, 0.92)	.000	0.87	0.00
GP: > 40	3	0.41 (0.05, 0.77)	.028	0.07	61.61
CN: < 40	4	0.64 (0.31, 0.97)	.000	0.13	45.62
CN: > 40	3	−0.05 (−0.20, 0.09)	.452	0.42	61.61
Th: < 40	4	0.18 (−0.21, 0.58)	.359	0.04	63.98
Th: > 40	3	−0.71 (−0.86, −0.57)	.000	0.94	0.00
Sex (male %) subgroup					
PUT: < 25%	4	0.46 (0.13, 0.78)	.006	0.14	44.93
PUT: > 25%	3	0.55 (0.41, 0.70)	.000	0.53	0.00
GP: < 25%	4	0.52 (0.18, 0.86)	.002	0.12	46.63
GP: > 25%	3	0.61 (0.46, 0.75)	.000	0.67	0.00
CN: < 25%	4	0.35 (−0.02, 0.72)	.057	0.08	56.96
CN: > 25%	3	0.39 (−0.29, 1.08)	.253	0.00	89.42
Th: < 25%	4	0.02 (−0.60, 0.65)	.937	0.00	89.91
Th: > 25%	3	−0.56 (−0.90, −0.23)	.001	0.10	57.56
Disease duration (year) subgroup					
PUT: < 9.6	3	0.69 (0.43, 0.95)	.000	0.69	0.00
PUT: > 9.6	4	0.41 (0.17, 0.65)	.000	0.22	34.51
GP: < 9.6	3	0.72 (0.46, 0.98)	.000	0.87	0.00
GP: > 9.6	4	0.45 (0.18, 0.72)	.001	0.15	44.53
CN: < 9.6	3	0.74 (0.38, 1.10)	.000	0.16	44.88
CN: > 9.6	4	−0.02 (−0.18, 0.14)	.62	0.41	6.19
Th: < 9.6	3	0.21 (−0.33, 0.75)	.43	0.02	76.47
Th: > 9.6	4	−0.57 (−0.92, −0.21)	.001	0.05	65.24

Abbreviations: CI, confidence interval; CN, caudate nucleus; GP, globus pallidus; PUT, putamen; QSM, quantitative susceptibility mapping; SMD, standard mean difference; Th, thalamus.

Age‐based subgroup analysis revealed an interesting pattern. In subgroup analyses stratified by age, the χ values in the PUT (SMD = 0.64, 95% CI = 0.41–0.88, *p* = .000, *I* ^2^ = 0.00%, *k* = 4), GP (SMD = 0.68, 95% CI = 0.44–0.92, *p* = .000, *I* ^2^ = 0.00%, *k* = 4), and CN (SMD = 0.64, 95% CI = 0.31–0.97, *p* = .000, *I* ^2^ = 45.62%, *k* = 4) were significantly higher in RRMS patients aged < 40 years than in HCs. Conversely, the thalamus (SMD = 0.18, 95% CI = −0.21–0.58, *p* = .359, *I* ^2^ = 63.98%, *k* = 4) showed a nonsignificant effect. However, in RRMS patients > 40 years old, only the GP (SMD = 0.41, 95% CI = 0.05–0.77, *p* = .028, *I* ^2^ = 61.61%, *k* = 3) and PUT (SMD = 0.38, 95% CI = 0.06–0.70, *p* = .021, *I* ^2^ = 55.31%, *k* = 3) showed significantly higher χ values compared to HCs, but not in the CN (SMD = −0.05, 95% CI = −0.20–0.09, *p* = .452, *I* ^2^ = 61.61%, *k* = 3). Notably, the thalamus showed a different trend; the χ values were significantly reduced (lower in patients than in HCs) in the > 40 years subgroup (SMD = −0.71, CI = −0.86 to −0.57, *p* = .00, *I* ^2^ = 0.00%, *k* = 3). In total, the CN and thalamus exhibited divergent patterns in RRMS patients aged > 40 years.

Sex‐based subgroup analysis indicated varying effects on χ values in the DGM structures. When samples were stratified by sex, RRMS patients had significantly higher χ values in the PUT and GP than HCs, regardless of whether the samples had < 25% or > 25% males. Sex‐based subgroup analysis also indicated that higher male percentages were correlated with increased χ values in the PUT (SMD = 0.55, 95% CI = 0.41–0.70, *p* = .000, *I* ^2^ = 0.00%, *k* = 3) and GP (SMD = 0.61, 95% CI = 0.46–0.75, *p* = .000, *I* ^2^ = 0.00%, *k* = 3). However, the CN group did not show any significant sex‐related differences. For the thalamus, χ values were significantly lower among samples with > 25% males (SMD = −0.56, 95% CI = −0.90 to −0.23; *p* = .001, *I* ^2^ = 57.56%, *k* = 3) but did not show significant sex‐related differences for < 25% (SMD = 0.02, 95% CI = −0.60–0.65, *p* = .937, *I* ^2^ = 89.91%, *k* = 4).

In subgroup analyses stratified by disease duration, the χ values in the PUT (SMD = 0.69, 95% CI = 0.43–0.95, *p* = .000, *I* ^2^ = 0.00%, *k* = 3), GP (SMD = 0.72, 95% CI = 0.46–0.98, *p* = .000, *I* ^2^ = 0.00%, *k* = 3), and CN (SMD = 0.74, 95% CI = 0.38–1.10, *p* = .000, *I* ^2^ = 44.88%, *k* = 3) were significantly higher in patients with RRMS disease duration lower than 9.6 years than in HCs. Interestingly, the thalamus (SMD = −0.57, 95% CI = −0.92 to −0.21, *p* = .001, *I* ^2^ = 65.24%, *k* = 4) showed a significantly lower QSM value in patients with RRMS than in HCs.

### 3.4 Publication Bias and Risk of Bias Assessment Results

**FIGURE 6:**
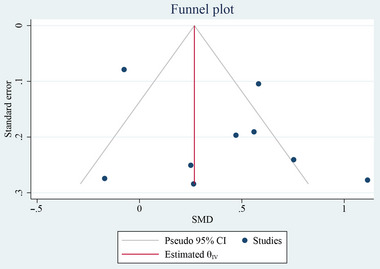
Funnel plot for publication bias with pseudo 95% confidence limits for RRMS studies.

We employed Egger's test and a funnel plot to evaluate the presence of publication bias, with a significance level of *p* < .05 indicating significant bias. The funnel plot displayed in Figure 6 indicates no publication bias, which is further supported by the results of the Egger's test (*p* = .852) (Figure S14). The quality of the included studies was assessed using NOS, as presented in Table S2. The average NOS score was 7.66 (range: 7–8), indicating good quality findings.

## 4 Discussion

Iron is an essential trace element that is involved in various biological processes, including oxygen transport, energy metabolism, and myelin synthesis. Excessive iron accumulation can lead to oxidative stress, inflammation, and neuronal damage (Doğan and Yildiz 2019; Stankiewicz, Neema, and Ceccarelli 2014). In general, increased χ values in the DGM structures of RRMS patients may contribute to the neurodegenerative processes and clinical symptoms observed in the disease. The χ values in the PUT, GP, and CN of RRMS patients have been found to increase, which is consistent with previous studies (Burgetova et al. 2017; Cobzas et al. 2015; Elkady et al. 2017, 2019; Hagemeier, Ramanathan, et al. 2018; Hagemeier, Zivadinov, et al. 2018; Khalil et al. 2015; Langkammer et al. 2013; Stankiewicz, Neema, and Ceccarelli 2014; Walsh et al. 2013; Zivadinov et al. 2018). This supports the hypothesis of iron dysregulation and increased iron accumulation in these DGM structures (Abo‐Krysha and Rashed 2008; Burgetova et al. 2022; Elkady et al. 2017; Neema et al. 2009; Rudko et al. 2014; Uddin et al. 2016). In addition, the iron concentration in the DGM is linked to the severity of clinical symptoms and can predict future disability (Dusek et al. 2022; Eskreis‐Winkler et al. 2017; Schmalbrock et al. 2016).

The QSM can show the bulk χ of diamagnetic and paramagnetic materials through negative and positive values as low‐ and high‐contrast images (Harada et al. 2022). Thus, a negative value (dark) indicates diamagnetic susceptibility, and a positive value (bright) indicates paramagnetic susceptibility (Ghaderi et al. 2024). These overall findings align with previous studies that have reported higher χ values (iron accumulation pattern) in the DGM structures of RRMS patients using various MRI techniques, including T2 and T2^*^‐weighted (Al‐Radaideh et al. 2019; Bakshi et al. 2002; Bermel et al. 2005; Du et al. 2015; Neema et al. 2009; Zhang et al. 2010), R2^*^ (Hernández‐Torres et al. 2019; Rudko et al. 2014; Walsh et al. 2014), phase data (Zivadinov et al. 2012), SWI (Habib et al. 2012), and histopathology (Haider et al. 2014). The observed dysregulation of iron in RRMS is thought to arise from a combination of factors. The inflammatory and demyelinating processes characteristic of RRMS can lead to the loss of axonal cells and myelin damage, which can disrupt iron homeostasis and promote iron accumulation (Papiri et al. 2023). Gradual demyelination can lead to an autoimmune response and a cycle of further degeneration, which is a characteristic of the most common form of MS (especially RRMS). According to this model, the autoimmune and/or inflammatory response is a secondary reaction to the primary process that causes death of oligodendrocytes and demyelination of axons in the CNS (Bamm and Harauz 2014; Stys et al. 2012). Furthermore, activated microglia, the resident macrophages of the brain, play a crucial role in phagocytosis of myelin debris and iron‐containing substances (Haider et al. 2014; Hamdy et al. 2022; Schweser et al. 2018). However, excessive microglial activation can contribute to iron dysregulation (Muzio, Viotti, and Martino 2021; Pukoli and Vécsei 2023). In addition, the blood–brain barrier (BBB), which normally restricts the movement of iron into the brain, can become compromised in RRMS, allowing for increased iron influx (Gillen et al. 2018; Khalil, Teunissen, and Langkammer 2011).

There was no significant difference in thalamic χ values between RRMS patients and HCs. Subgroup analyses revealed reduced χ values in patients over 40 years of age and in those with a higher male population. This confirms the consistent findings between previous studies that reported decreased iron levels in the thalamus of patients with RRMS (Bergsland et al. 2018; Burgetova et al. 2017; Khalil et al. 2015; Pontillo et al. 2019; Pudlac et al. 2020; Schweser et al. 2018, 2020; Stankiewicz, Neema, and Ceccarelli 2014; Walsh et al. 2013; Zivadinov et al. 2018). This decrease in susceptibility may be attributed to a lower presence of paramagnetic material or a higher presence of diamagnetic myelin production. However, myelin content is unlikely to be higher in MS (Burgetova et al. 2017; Langkammer et al. 2012). Therefore, our finding of negative thalamic susceptibility values in patients with RRMS aligns more with the loss of iron content (Burgetova et al. 2017).

One study found that individuals with RRMS had higher iron levels in the PUT and CN than those with CIS (Khalil et al. 2009). Another cross‐sectional study also showed that even in early RRMS, iron accumulates in the DGM, which may explain the natural progression of RRMS to SPMS (Al‐Radaideh et al. 2019). These studies align with our findings and provide insights into the conversion of less advanced MS phenotypes, such as CIS to RRMS and RRMS to SPMS. However, the relationship between iron deposition and disease progression from CIS to RRMS and RRMS to SPMS remains unclear.

It is interesting to note that χ values in the brain are influenced by age and sex, as shown by subgroup analyses. Younger patients with RRMS (those under 40 years) had higher χ values in the GP, CN, and PUT compared to HCs. This finding is consistent with that of a previous study (Habib et al. 2012) and suggests that iron dysregulation may play a role in the early stages of the disease (Rudko et al. 2014). However, both younger and older RRMS patients had increased χ values in the PUT and GP but not in the CN or thalamus in the older age group. This may indicate region‐specific vulnerability or compensatory response that occurs with advancing age (Correale, Marrodan, and Ysrraelit 2019; Sandi et al. 2021). The QSM values of the thalamus showed a different trend according to age; older RRMS patients had lower χ values than younger RRMS patients. This is consistent with previous studies showing age‐related decreases in iron levels in the thalamus of healthy individuals (Bergsland et al. 2018; Burgetova et al. 2017; Khalil et al. 2015; Pontillo et al. 2019; Pudlac et al. 2020; Schweser et al. 2018, 2020; Stankiewicz, Neema, and Ceccarelli 2014; Walsh et al. 2013; Zivadinov et al. 2018). For example, Schweser et al. (2020) noted that MS patients had significantly lower iron content in the thalamus than HCs, with progressive MS patients demonstrating lower iron content than RRMS, which aligns with our findings. Our results also align with recent studies suggesting that older RRMS patients may lack compensatory abilities, which could explain the decrease in thalamic iron content (Bergsland et al. 2016; Pontillo et al. 2019; Schweser et al. 2018). This reduction in χ values may be due to selective neuronal loss, atrophy, iron deficiency, and demyelination at different stages of MS (Andravizou et al. 2019; Haider et al. 2014; Sandi et al. 2021). However, the mechanisms and implications of iron depletion in the thalamus require further investigation.

Furthermore, based on disease duration subgroup analysis, it seems the rate of QSM value increased in the early stages of the patients with RRMS in the PUT, GP, and CN. While in the thalamus, QSM value tends to decrease in the later stages of the patients than in the HCs. Thus, we recommend conducting future longitudinal studies to validate these findings.

While the current literature often attributes increased QSM values to iron accumulation (Cogswell et al. 2021), it is crucial to exercise caution when interpreting these values to the potential atrophy of DGM structures. Addressing DGM atrophy is a vital consideration when discussing QSM within these regions. Recent studies have proposed that the frequently observed elevation in DGM iron levels in MS patients may be partly due to the loss of cells with minimal or no iron content (Schweser et al. 2020). These insights provide a new perspective on the emerging imaging research that has primarily supported the idea that neurodegeneration in MS (Pontillo et al. 2021; Schweser et al. 2020) and other neurodegenerative diseases (Chen et al. 2024; Ravanfar et al. 2021) is associated with iron influx into the DGM. Consequently, prior literature advises that variations in regional tissue iron concentrations should be interpreted within the appropriate context of structural volume changes, such as alongside an iron‐content metric.

It is important to note that the current approach of studying the thalamus as a single unit in MS research may be misleading. The thalamus is a complex structure with numerous distinct subregions, each with different functions and connections (Iglesias et al. 2018). Neuroimaging studies have shown that specific areas within the thalamus are more affected by MS than others (Bisecco et al. 2021; Minagar et al. 2013). By analyzing the whole thalamus together, we might be missing this crucial detail and obtaining an averaged picture that does not reflect the true heterogeneity of the disease impact.

We found that males had higher χ values, particularly in the PUT and GP regions. This could be due to biological differences or lifestyle factors that affect iron metabolism in patients (Grubić Kezele and Ćurko‐Cofek 2020). This finding is consistent with previous studies that reported higher iron levels in males than females in the general population (Bartzokis et al. 1997, 2010; Hagemeier, Ramanathan, et al. 2018). The differential effects of sex may indicate the influence of sex hormones on iron regulation, which may modulate the subcortical vulnerability in RRMS. Estrogen, for instance, has antioxidant and neuroprotective effects that can mitigate iron‐catalyzed oxidative damage (Torrens‐Mas et al. 2020). This finding also highlights the complexities of the pathophysiological processes underlying MS and emphasizes the need for sex‐sensitive approaches in research and treatment strategies.

Sex‐based subgroup analysis revealed divergent χ values in the DGM structures, which reiterates the pivotal impact of sex on MS pathology. Our analysis revealed elevated iron levels in the PUT and GP, regardless of sex. This is in agreement with previous studies that reported no sex differences in GM atrophy in with RRMS patients (Antulov et al. 2009; Dolezal et al. 2013). However, an interesting trend emerged with the thalamic χ values, which were notably reduced among samples with a male predominance (Burgetova et al. 2017; Langkammer et al. 2013; Zivadinov et al. 2018), possibly reflecting differing disease progression and susceptibility patterns across sexes. Sex differences in iron levels may be related to hormonal, genetic, or environmental factors that influence iron metabolism and homeostasis (Hagemeier, Ramanathan, et al. 2018; Lury et al. 2023).

The studies included in the research showed some inconsistencies in χ values, for example, in the thalamus, between patients with RRMS and HCs. Cobzas et al. (2015) and Pudlac et al. (2020) showed higher χ values in RRMS patients, while others, such as Burgetova et al. (2017), Hagemeier, Ramanathan et al. (2018), Hagemeier, Zivadinov, et al. (2018), and Zivadinov et al. (2018) found lower χ values in the thalamus of RRMS patients than in HCs. These inconsistencies are apparent in Table 1, which ranges from negative values (as in Hagemeier, Ramanathan, et al. (2018), Hagemeier, Zivadinov, et al. (2018), and Zivadinov et al. (2018)) to values exceeding 70 (as in (Cobzas et al. 2015). These differences may be attributed to variations in the analytical methods used for QSM across the studies. For example, Cobzas et al. (2015) and Elkady et al. (2017, 2019) used the regularization‐abled sophisticated harmonic artifact reduction for phase data (RESHARP) method for background field removal and phase unwrapping, followed by dipole inversion for QSM map creation. Conversely, Hagemeier, Ramanathan, et al. (2018), Hagemeier, Zivadinov, et al. (2018), and Zivadinov et al. (2018) opted for the variable‐kernel sophisticated harmonic artifact reduction for phase data (V‐SHARP) technique for background field removal, along with the best path algorithm for phase unwrapping, using the Homogeneity Enabled Dipole Inversion (HEIDI) method for map reconstruction. Burgetova et al. (2017) and Pudlac et al. (2020), on the other hand, applied total generalized variation for all stages of map creation. The discrepancies in the results can be primarily attributed to the different post‐processing techniques used for QSM reconstruction, which can affect the susceptibility distributions and create systematic deviations in the extracted QSM values (Ghaderi et al. 2023; Ravanfar et al. 2021). Standardization of crucial processing steps, such as background field removal, phase‐unwrapping approaches tailored to DGM structures, and validated QSM reconstruction methods, can improve reproducibility across studies (Committee et al. 2024).

Heterogeneity results in the meta‐analysis revealed variability across brain regions. While all regions except the GP (I^2^ = 0.00 %) showed moderate‐to‐high heterogeneity (I^2^ = 56.92%–88.74%, *p* values .00–.06), further exploration is warranted due to the inconsistency. Subgroup analysis also seems to exhibit variable heterogeneity, suggesting potential moderating factors that require further investigation. This highlights the importance of exploring potential sources of heterogeneity to strengthen the interpretation of overall effect sizes.

Further research should focus on understanding the underlying mechanisms of iron dysregulation in RRMS and exploring the potential therapeutic benefits of targeting iron homeostasis. The investigation of iron‐modulating therapies in relation to various factors such as age, sex, and disease duration has the potential to expand available treatment options. Additionally, conducting prospective studies that assess iron levels over time, in conjunction with disease progression and treatment responses, could provide valuable insights for managing MS. The findings were strengthened by the absence of publication bias and the high quality of the included studies, as indicated by the average NOS score. However, the use of different magnetic field strengths, coils, and QSM analysis methods in the included studies may have introduced heterogeneity in the results. Furthermore, the studies were conducted in North America and Europe, which may limit the generalizability of the findings. Nevertheless, by pooling QSM data from multiple studies, this meta‐analysis provides evidence that supports an association between pathological iron accumulation and subcortical GM pathology in RRMS. Owing to limited data availability, the potential impact of disease duration, medication, and other clinical factors on iron accumulation was not assessed.

## 5 Conclusions

In summary, this systematic review and meta‐analysis provides insights into the patterns of alteration in χ values, suggesting iron accumulation in specific regions of the DGM in patients with RRMS. The main findings reveal that RRMS patients had significantly higher χ values in the PUT, GP, and CN than in HCs. Subgroup analysis based on age and sex shows that younger patients (< 40 years) had more significant χ values in the PUT, GP, and CN and that a larger male population in the PUT and GP (> 25%) had more significant χ values. Interestingly, χ values in the thalamus decreased with RRMS patients over 40 years old and those with a higher male population. Sex‐based analysis indicates higher χ values in the PUT and GP of RRMS patients, regardless of sex. We found that QSM values increased more in the early stages (disease duration < 9.6 years) of RRMS in certain brain regions (PUT, GP, and CN) but decreased in the thalamus in later stages (disease duration > 9.6 years) than HCs. Further longitudinal research is needed to confirm these findings.

## Author Contributions

**Sana Mohammadi**: conceptualization, methodology, software, formal analysis, data curation, supervision, project administration, visualization, writing–review and editing, writing–original draft, investigation, validation. **Sadegh Ghaderi**: conceptualization, investigation, writing–original draft, writing–review and editing, visualization, validation, methodology, software, formal analysis, project administration, supervision, data curation. **Farzad Fatehi**: conceptualization, methodology, validation, writing–review and editing, writing–original draft, supervision, project administration.

## Conflicts of Interest

The authors declare no conflicts of interest.

### Peer Review

The peer review history for this article is available at https://publons.com/publon/10.1002/brb3.70093

## Supporting information



Supplementary Materials.

## Data Availability

Data supporting the findings of this study are available upon request from the corresponding author.
