# Supplementary material for: Quantitative Susceptibility Mapping Values Quantification in Deep Gray Matter Structures for Relapsing‐Remitting Multiple Sclerosis: A Systematic Review and Meta‐Analysis
Source: Brain Behav. 2024 Oct 17;14(10):e70093. doi: 10.1002/brb3.70093 (PMC11483550; doi:10.1002/brb3.70093)
Supplement: Supplementary file 1 — Supplementary Materials. [file BRB3-14-e70093-s001.docx]

**
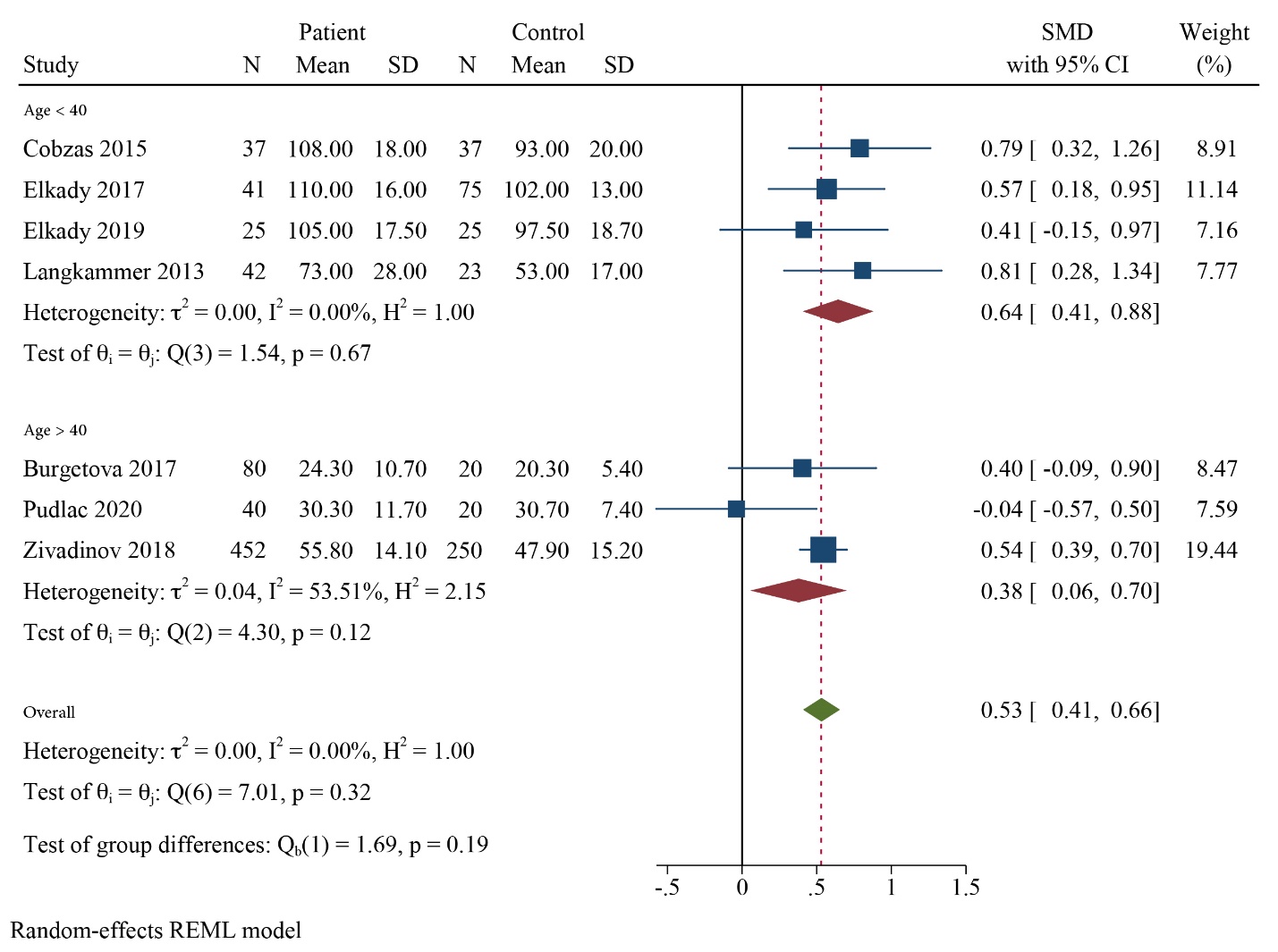
**

### **Supplementary Figure 1.** Subgroup analysis of age-related changes for putamen (<40 or >40 years)

###
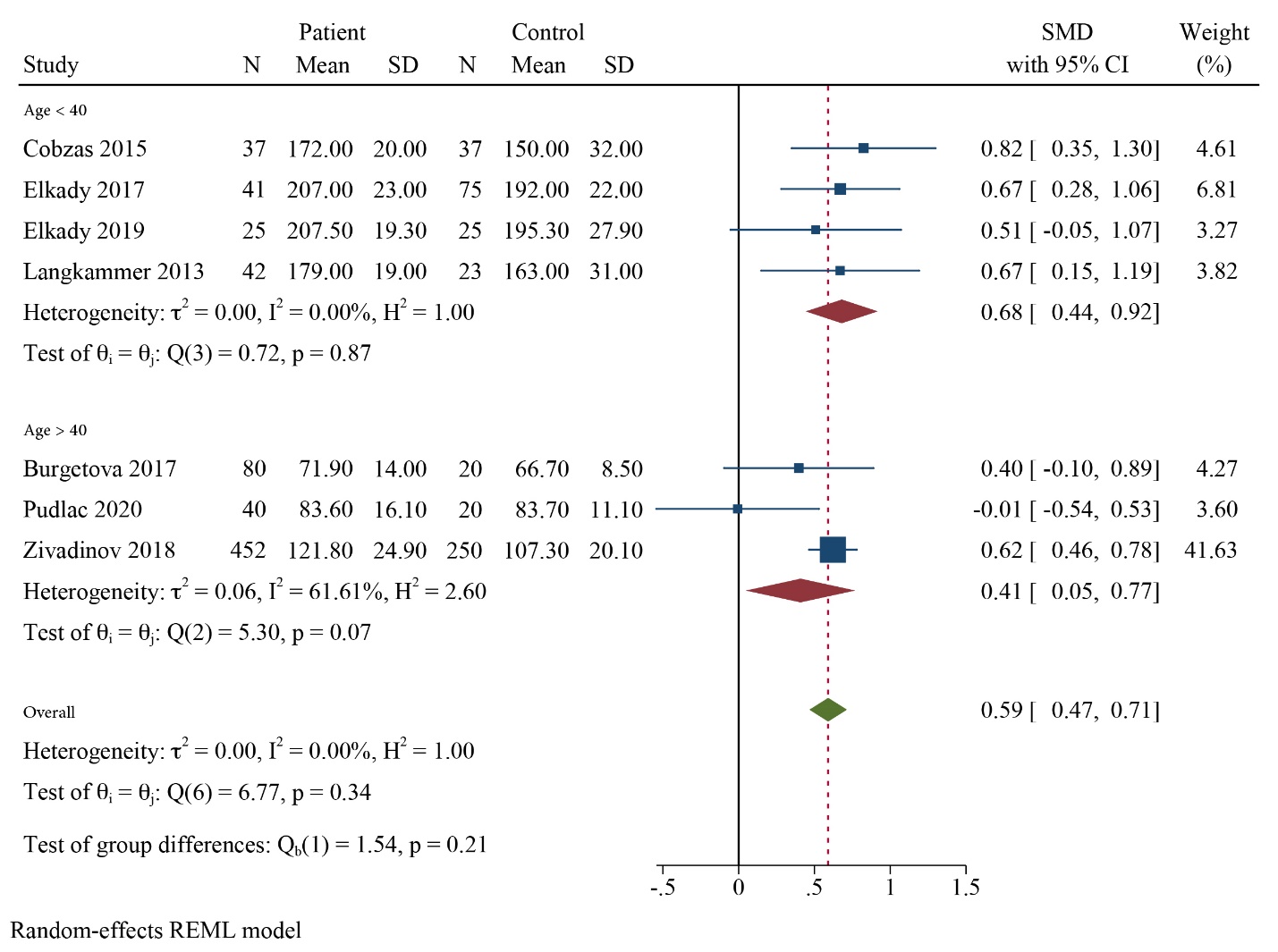


### **Supplementary Figure 2.** Subgroup analysis of age-related changes for globus pallidus (<40 or >40 years)

###
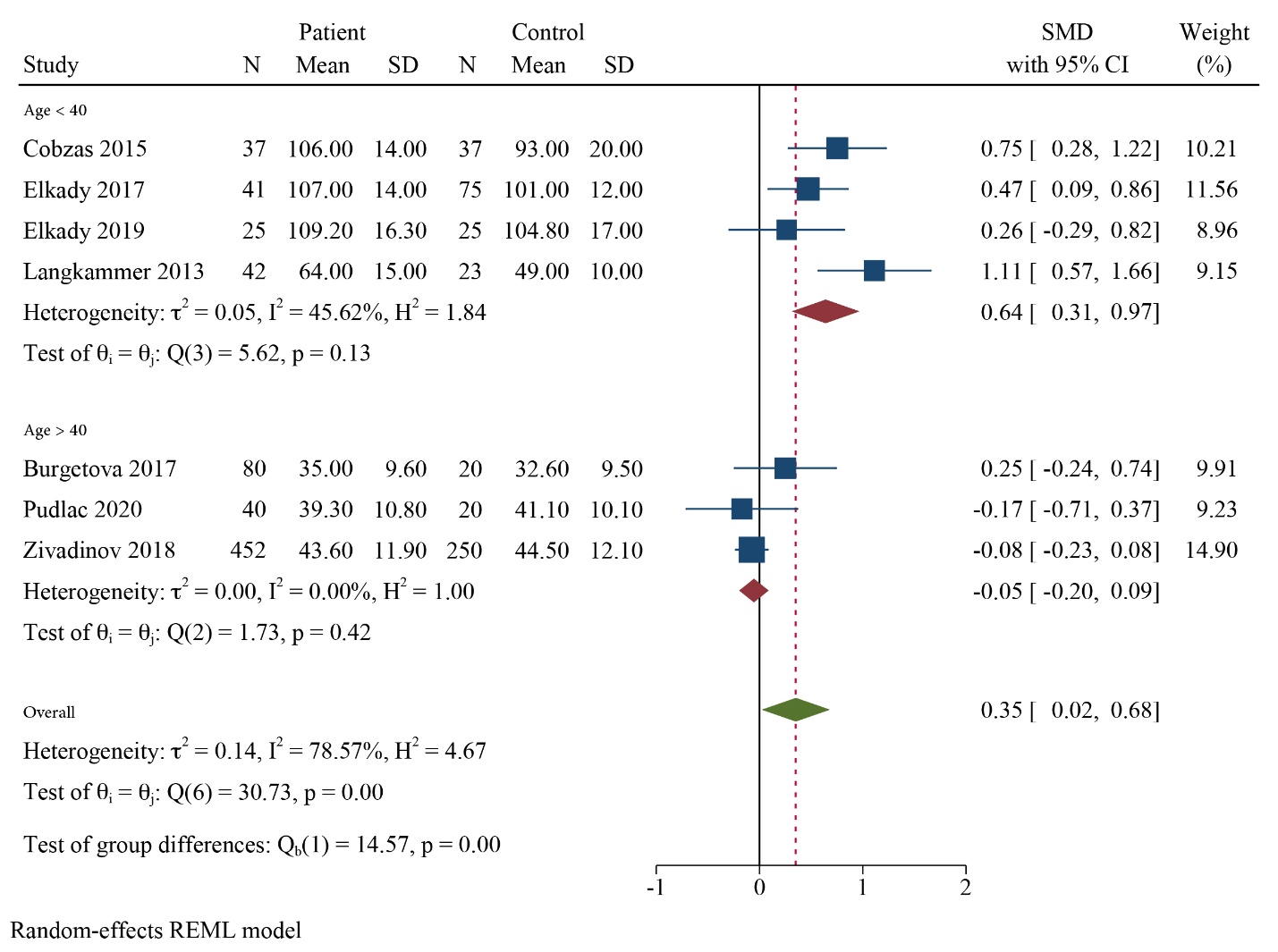


### **Supplementary Figure 3.** Subgroup analysis of age-related changes for caudate nucleus (<40 or >40 years)

###
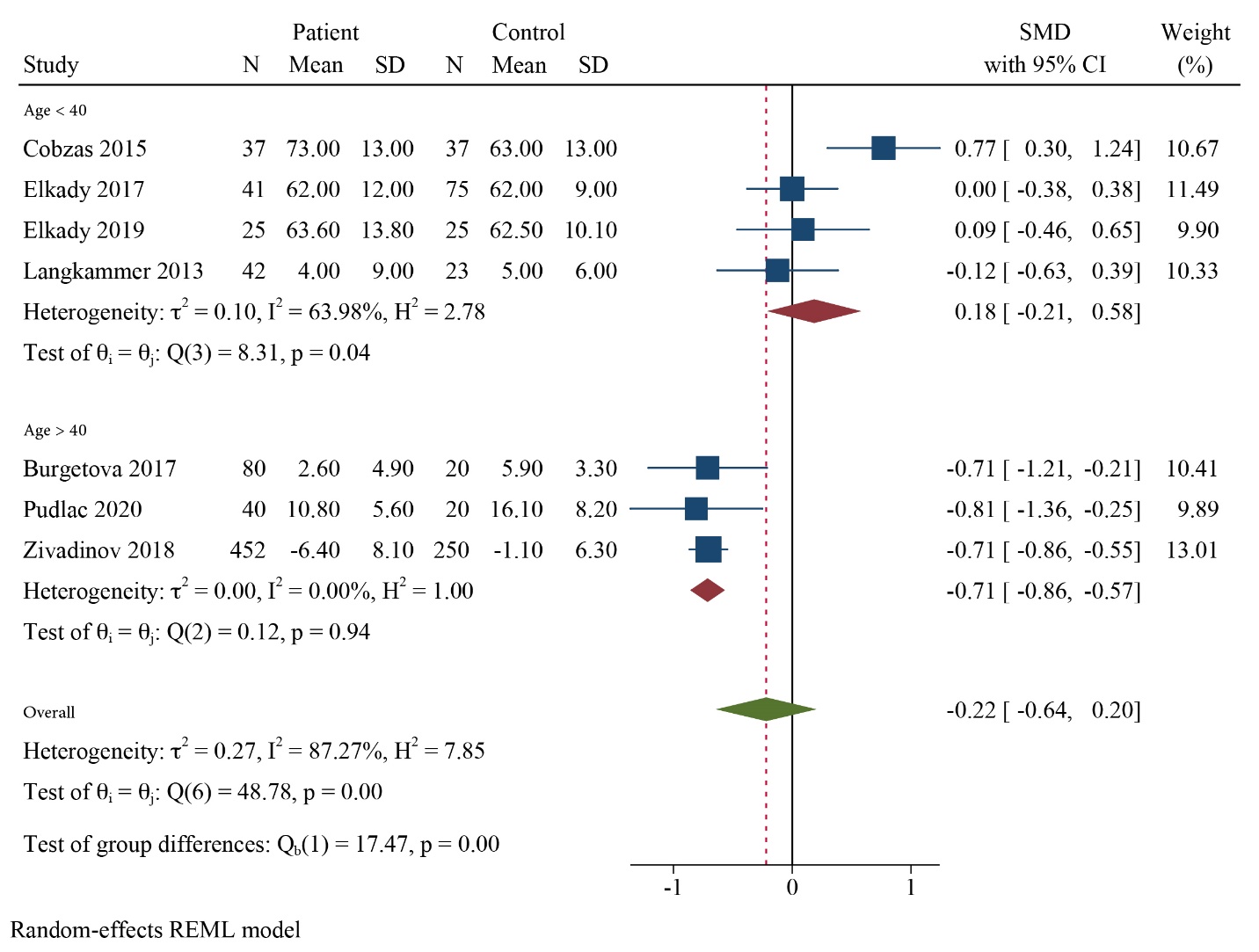


### **Supplementary Figure 4.** Subgroup analysis of age-related changes for thalamus (<40 or >40 years)

###
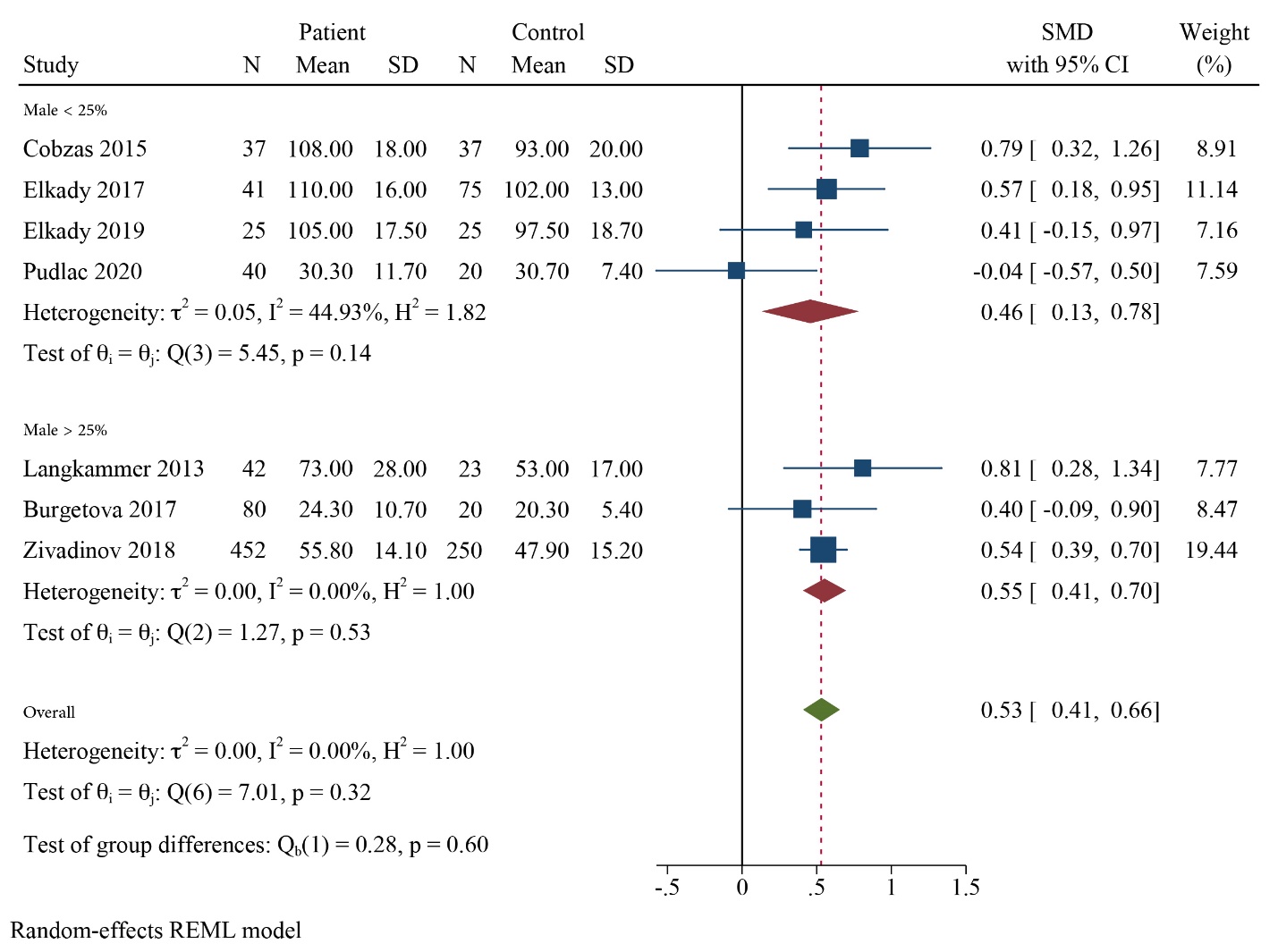


### **Supplementary Figure 5.** Subgroup analysis of sex changes for putamen (<25% or >25% males)

###
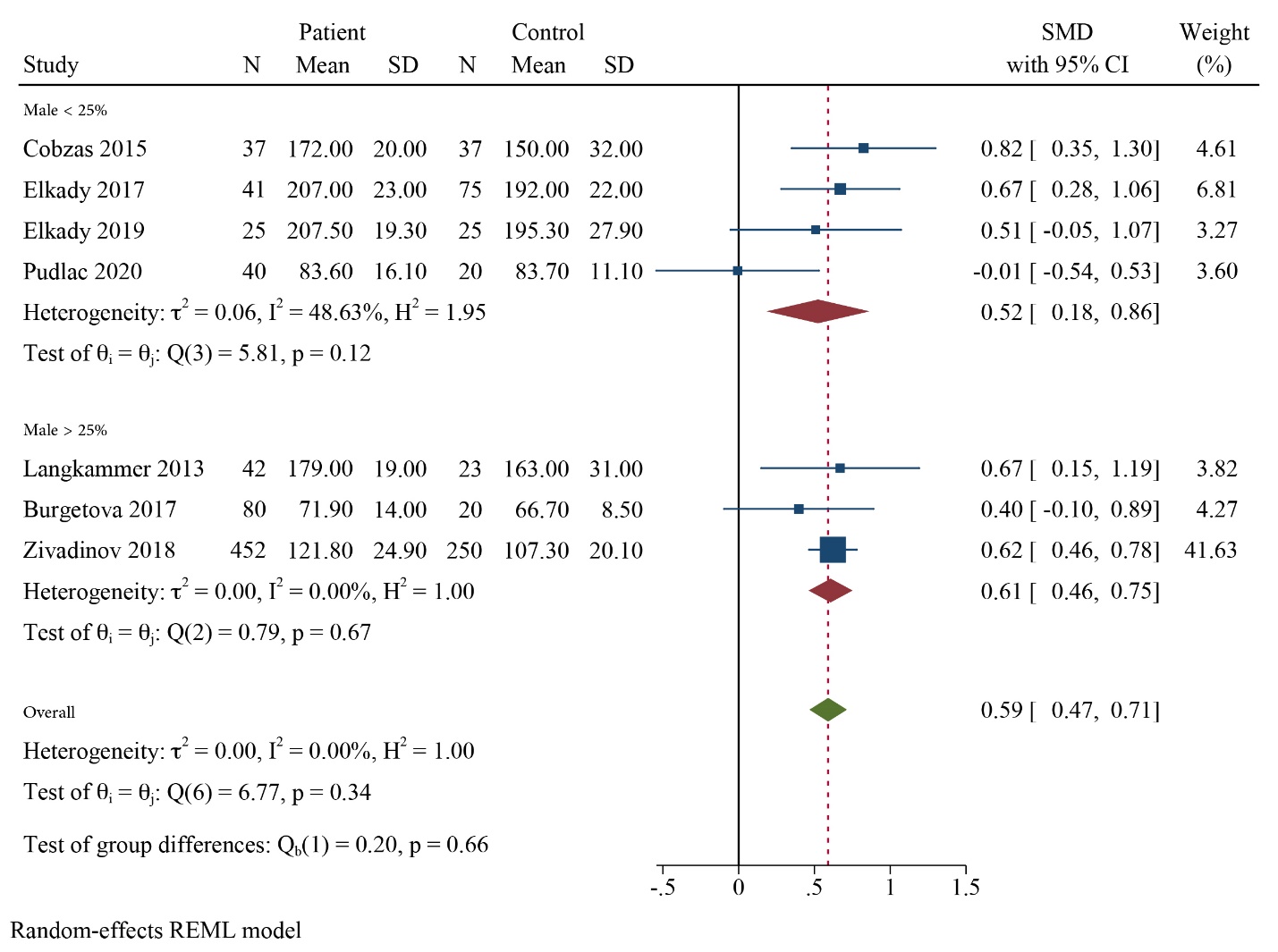


### **Supplementary Figure 6.** Subgroup analysis of sex changes for globus pallidus (<25% or >25% males)

###
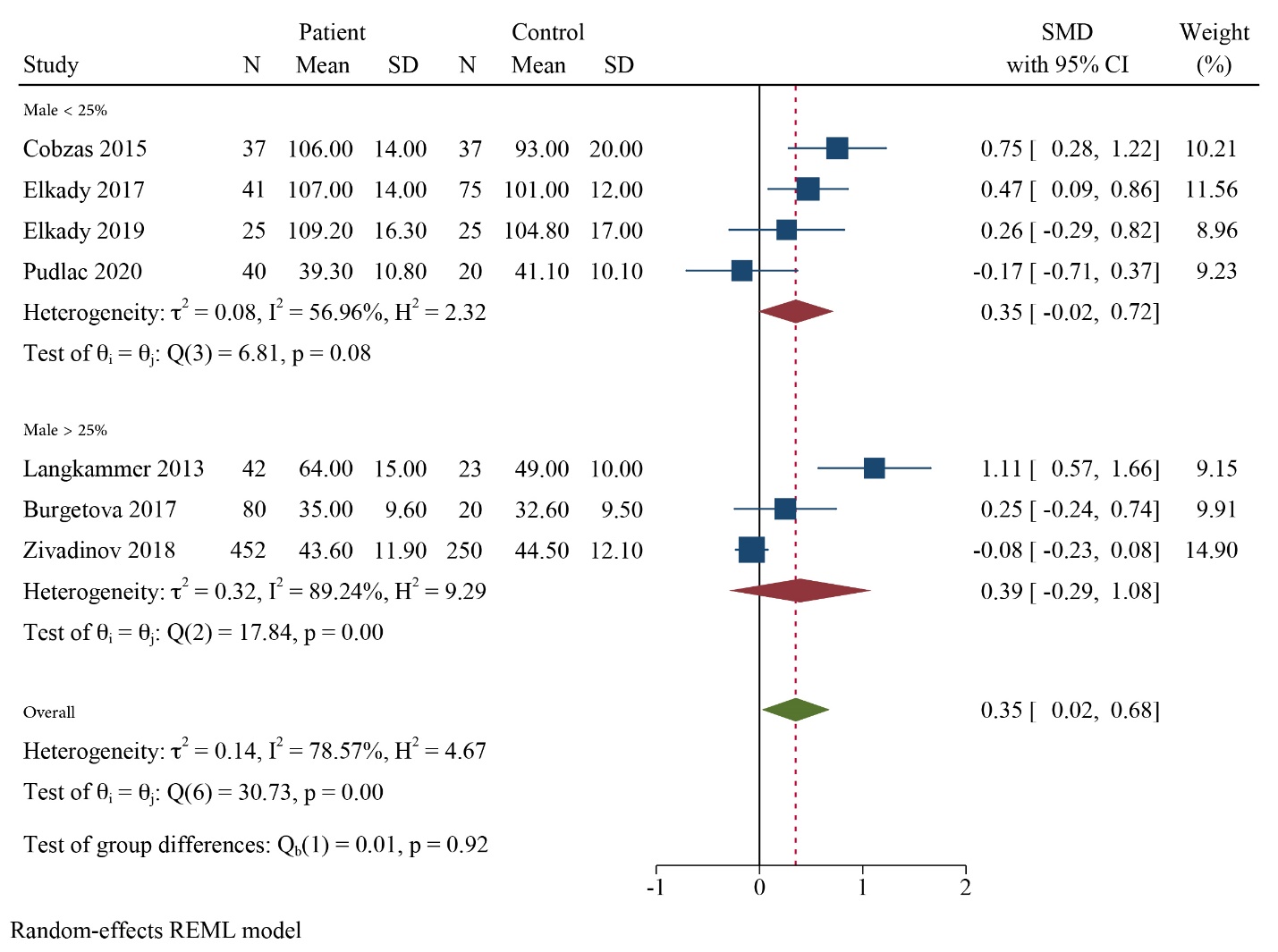


### **Supplementary Figure 7.** Subgroup analysis of sex changes for caudate nucleus (<25% or >25% males)

###
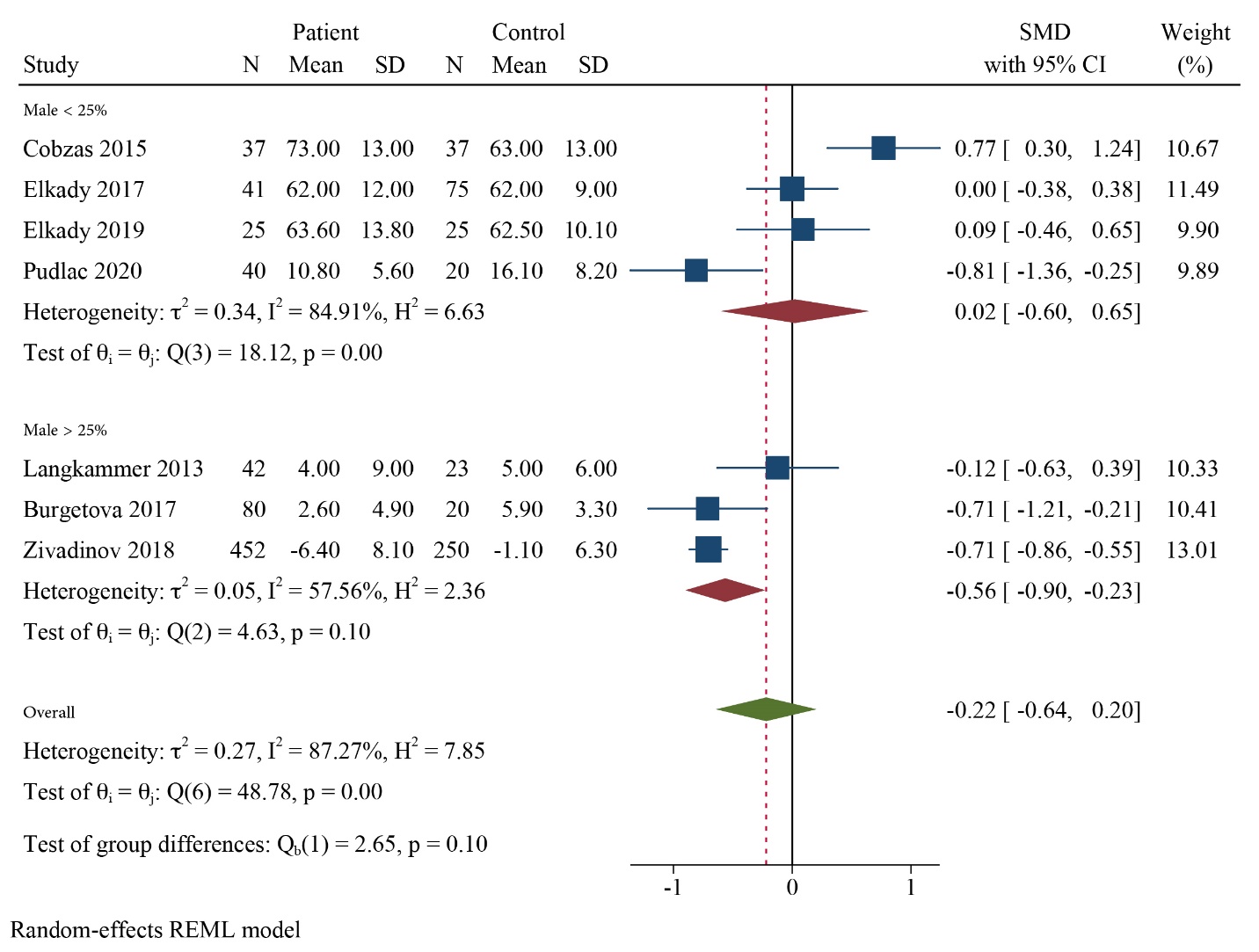


### **Supplementary Figure 8.** Subgroup analysis of sex changes for thalamus (<25% or >25% males)

###
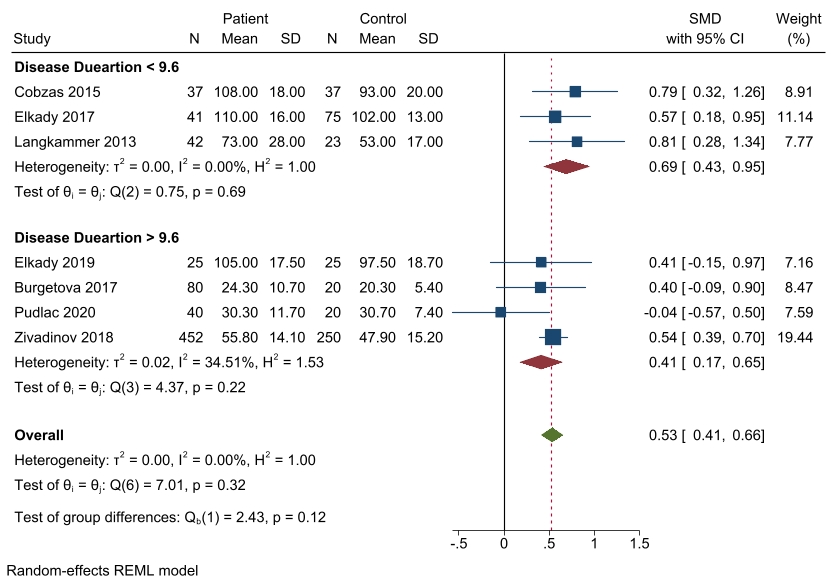


**Supplementary Figure 9.** Subgroup analysis of disease duration changes for putamen (<9.6 or >9.6 years)

**
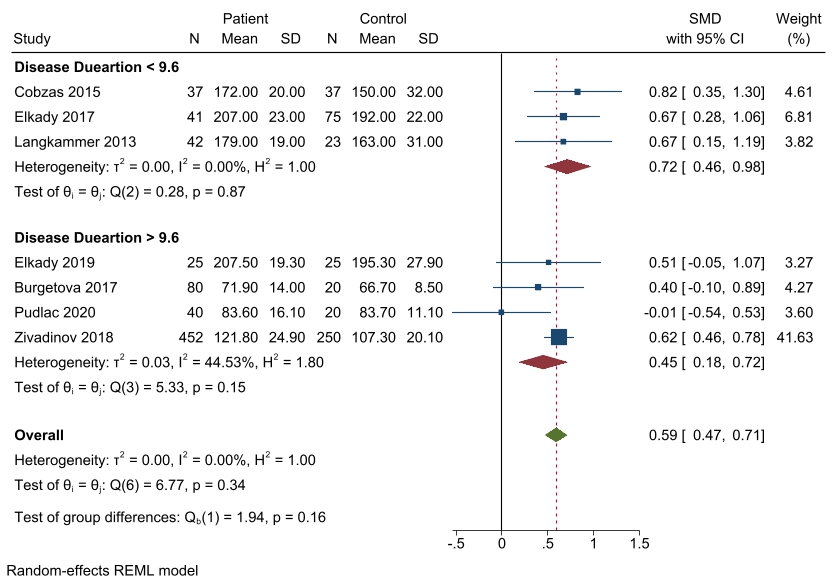
**

**Supplementary Figure 10.** Subgroup analysis of disease duration changes for globus pallidus (<9.6 or >9.6 years)

**
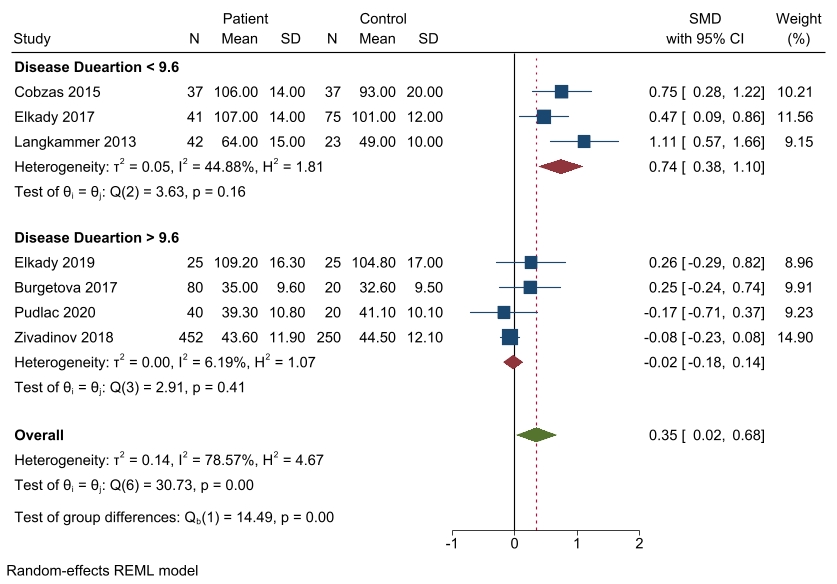
**

**Supplementary Figure 11.** Subgroup analysis of disease duration changes for caudate nucleus (<9.6 or >9.6 years)

**
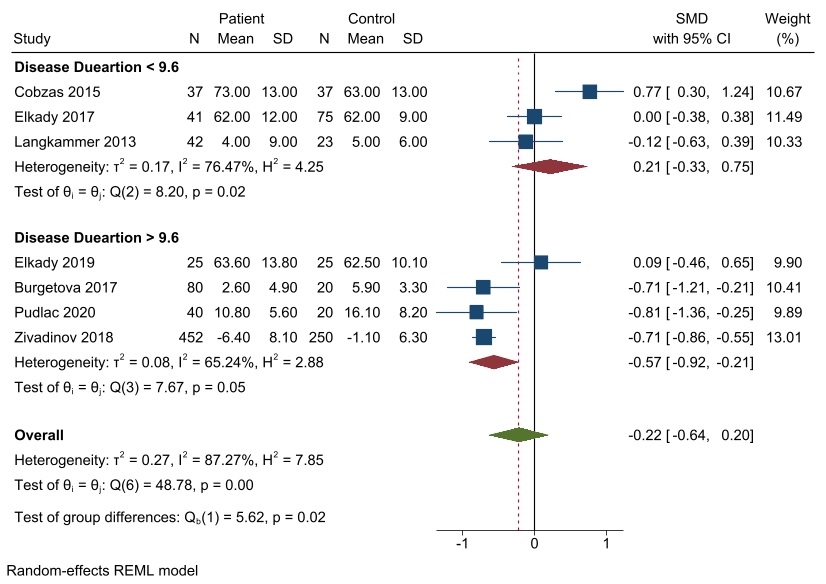
**

**Supplementary Figure 12.** Subgroup analysis of disease duration changes for thalamus (<9.6 or >9.6 years)

###
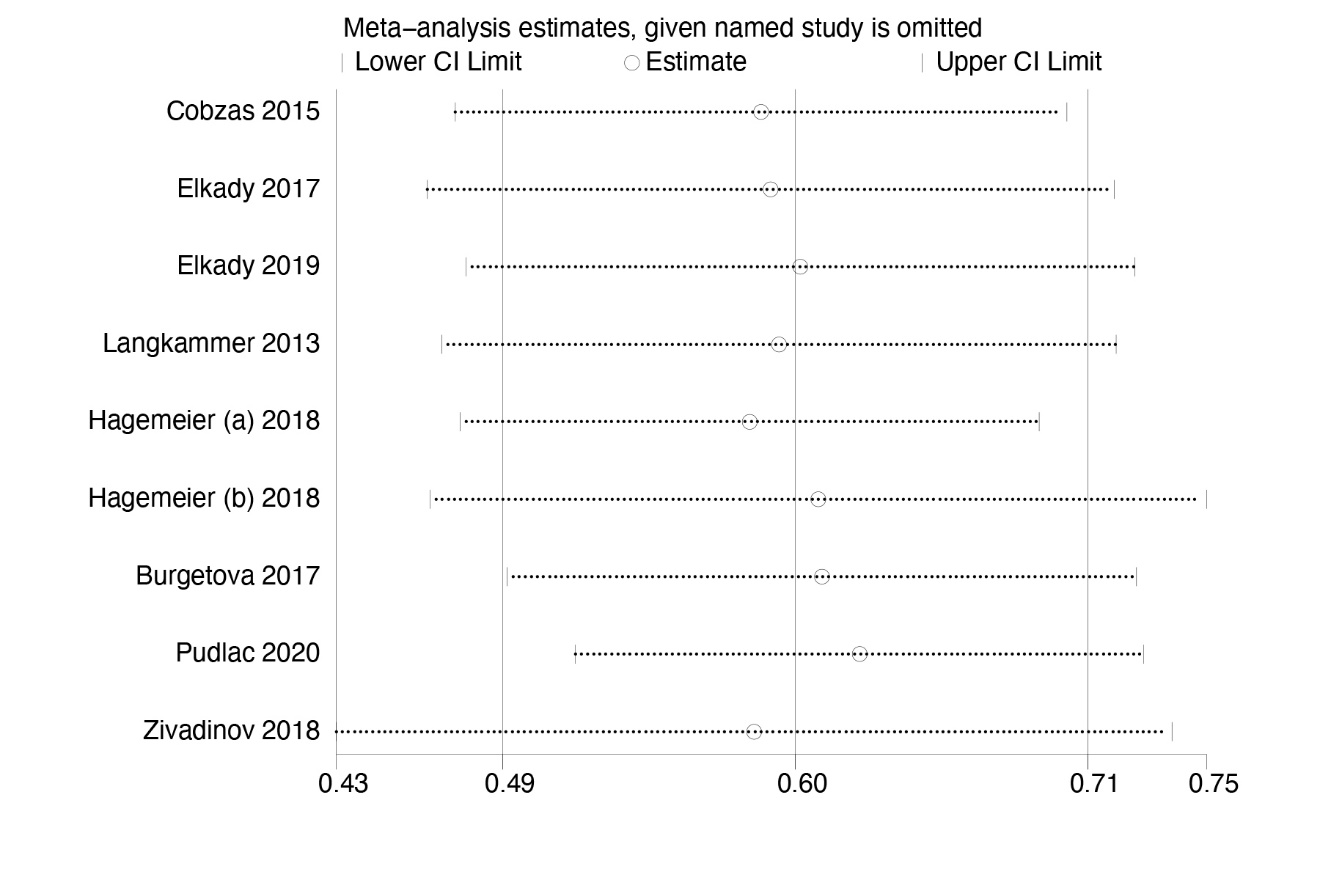


**Supplementary Figure 13.** Sensitivity analysis of included studies


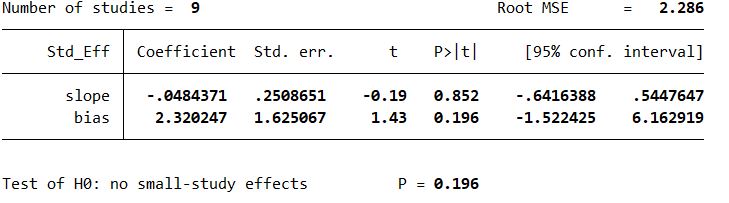


**Supplementary Figure 14.** Egger’s test results

| **Supplementary Table 1.** The search strategies used for database searches | |
| --- | --- |
| **Database** | **N** |
| PubMed: (("relapsing-remitting multiple sclerosis" OR "multiple sclerosis") AND ("quantitative susceptibility Mapping" OR "QSM") AND ("Basal ganglia" OR "Striatum" OR "caudate nucleus" OR "putamen" OR "globus pallidus" OR "substantia nigra pars reticulata" OR "subthalamic nucleus" OR "thalamus" OR "red nucleus" OR "substantia nigra pars compacta" OR "substantia nigra")) | 32 |
| Scopus: (TITLE-ABS-KEY("relapsing-remitting multiple sclerosis" OR "multiple sclerosis") AND TITLE-ABS-KEY("quantitative susceptibility Mapping" OR "QSM") AND TITLE-ABS-KEY("basal ganglia" OR "striatum" OR "caudate nucleus" OR "putamen" OR "globus pallidus" OR "substantia nigra pars reticulata" OR "subthalamic nucleus" OR "thalamus" OR "red nucleus" OR "substantia nigra pars compacta" OR "substantia nigra")) | 46 |
| Web of Science: TS=(("relapsing-remitting multiple sclerosis" OR "multiple sclerosis") AND ("quantitative susceptibility Mapping" OR "QSM") AND ("basal ganglia" OR "striatum" OR "caudate nucleus" OR "putamen" OR "globus pallidus" OR "substantia nigra pars reticulata" OR "subthalamic nucleus" OR "thalamus" OR "red nucleus" OR "substantia nigra pars compacta" OR "substantia nigra")) | 79 |

| **Supplementary Table 2.** Qulity assessments results (the Newcastle-Ottawa Scale adapted to the cross-sectional studies) | | | | | | | | |
| --- | --- | --- | --- | --- | --- | --- | --- | --- |
| **Author (year)** | **Selection** | | | | **Comparability** | **Outcome** | | **Total score** |
|  | **Representativeness of the sample** | **Sample size** | **Non-respondents** | **Ascertainment of the exposure (risk factor)** | **Control for important or additional factors** | **Assessment of the outcome** | **Statistical test** |  |
| Pudlac (2020) ^1^ | ***** | ***** |  | ****** | ***** | ***** | ***** | 7 |
| Elkady (2019) ^2^ | ***** | ***** | ***** | ****** | ***** | ***** | ***** | 8 |
| Hagemeier (a) (2018) ^3^ | ***** | ***** | ***** | ****** |  | ***** | ***** | 7 |
| Hagemeier (b) (2018) ^4^ | ***** | ***** | ***** | ****** |  | ***** | ***** | 7 |
| Burgetova (2017) ^5^ | ***** | ***** | ***** | ****** | ***** | ***** | ***** | 8 |
| Elkady (2017) ^6^ | ***** | ***** | ***** | ****** | ***** | ***** | ***** | 8 |
| Cobzas (2015) ^7^ | ***** | ***** | ***** | ****** | ***** | ***** | ***** | 8 |
| Langkammer (2013) ^8^ | ***** | ***** | ***** | ****** | ***** | ***** | ***** | 8 |
| Zivadinov (2018) ^9^ | ***** | ***** | ***** | ****** | ***** | ***** | ***** | 8 |
|  | **Mean** | | | | | | | 7.66 |

**References**

1. Pudlac, A. *et al.* Deep Gray Matter Iron Content in Neuromyelitis Optica and Multiple Sclerosis. *Biomed Res Int* **2020**, 6492786 (2020).

2. Elkady, A. M. *et al.* Five year iron changes in relapsing-remitting multiple sclerosis deep gray matter compared to healthy controls. *Mult Scler Relat Disord* **33**, 107–115 (2019).

3. Hagemeier, J. *et al.* Changes of deep gray matter magnetic susceptibility over 2 years in multiple sclerosis and healthy control brain. *Neuroimage Clin* **18**, 1007–1016 (2018).

4. Hagemeier, J. *et al.* Iron-related gene variants and brain iron in multiple sclerosis and healthy individuals. *Neuroimage Clin* **17**, 530–540 (2018).

5. Burgetova, A. *et al.* Thalamic Iron Differentiates Primary-Progressive and Relapsing-Remitting Multiple Sclerosis. *AJNR Am J Neuroradiol* **38**, 1079–1086 (2017).

6. Elkady, A. M., Cobzas, D., Sun, H., Blevins, G. & Wilman, A. H. Progressive iron accumulation across multiple sclerosis phenotypes revealed by sparse classification of deep gray matter. *J Magn Reson Imaging* **46**, 1464–1473 (2017).

7. Cobzas, D. *et al.* Subcortical gray matter segmentation and voxel-based analysis using transverse relaxation and quantitative susceptibility mapping with application to multiple sclerosis. *J Magn Reson Imaging* **42**, 1601–1610 (2015).

8. Langkammer, C. *et al.* Quantitative susceptibility mapping in multiple sclerosis. *Radiology* **267**, 551–559 (2013).

9. Zivadinov, R. *et al.* Brain Iron at Quantitative MRI Is Associated with Disability in Multiple Sclerosis. *Radiology* **289**, 487–496 (2018).
